# Neutralizing antibodies for orthobunyaviruses in Pantanal, Brazil

**DOI:** 10.1371/journal.pntd.0006014

**Published:** 2017-11-01

**Authors:** Alex Pauvolid-Corrêa, Zilca Campos, Raquel Soares, Rita Maria Ribeiro Nogueira, Nicholas Komar

**Affiliations:** 1 Arbovirus Diseases Branch, Centers for Disease Control and Prevention (CDC), Fort Collins, CO, United States of America; 2 Embrapa Pantanal, Ministério da Agricultura Pecuária e Abastecimento, Corumbá, MS, Brasil; 3 Laboratório de Flavivírus, Instituto Oswaldo Cruz, Fundação Oswaldo Cruz (Fiocruz), Ministério da Saúde, Rio de Janeiro, RJ, Brasil; University of Texas Medical Branch, UNITED STATES

## Abstract

The Pantanal is a hotspot for arbovirus studies in South America. Various medically important flaviviruses and alphaviruses have been reported in domestic and wild animals in the region. To expand the knowledge of local arbovirus circulation, a serosurvey for 14 Brazilian orthobunyaviruses was conducted with equines, sheep and free-ranging caimans. Sera were tested for specific viral antibodies using plaque-reduction neutralization test (PRNT). Monotypic reactions were detected for Maguari, Xingu, Apeu, Guaroa, Murutucu, Oriboca, Oropouche and Nepuyo viruses. Despite the low titers for most of the orthobunyaviruses tested, the detection of monotypic reactions for eight orthobunyaviruses suggests the Pantanal as a region of great orthobunyavirus diversity. The present data, in conjunction with previous studies that detected a high diversity of other arboviruses, ratify the Pantanal as an important natural reservoir for sylvatic and medically important arboviruses in Brazil.

## Author summary

In the present study, we report the evidence of various orthobunyaviruses of medical importance in domestic and wild animals of the Pantanal, a large floodplain located in West-Central Brazil. Although various other arboviruses as flaviviruses and alphaviruses have been reported in the region, orthobunyaviruses are not commonly investigated in Pantanal. Positive results for eight orthobunyaviruses, including Maguari, Xingu, Apeu, Guaroa, Murutucu, Oriboca, Oropouche and Nepuyo viruses were detected in equines, sheep and caimans in the region. The findings reported here suggest the Pantanal as a region of great orthobunyavirus diversity and ratify the Pantanal as hotspot for arbovirus studies in South America.

## Introduction

The *Bunyaviridae* family contains over 300 enveloped viruses divided into five genera, including *Orthobunyavirus*, *Phlebovirus* and *Nairovirus*, which include arboviruses, viruses capable of alternately replicating in vertebrates and arthropods [1]. Orthobunyaviruses are classified into immunologically distinct groups based on their immunological relationships, including Bunyamwera, Bwamba, group C, Guama, Simbu, Mapputta, and California. Little is known of the pathogenicity of some of these groups for human beings or of the clinical manifestations of the associated disease [2]. In Brazil, dozens of orthobunyaviruses have been isolated including Oropouche virus (OROV), the most medically important orthobunyavirus in the country involved in explosive human outbreaks mainly in rural areas of northern Brazil [3]. Recently, OROV has also been reported in urban areas of the west-central region of the country [4].

The west-central region of Brazil encompasses the states of Goiás, Mato Grosso (MT), Mato Grosso do Sul (MS), and the Federal District and is the second largest region in the country. Located within the west-central region of Brazil is the Pantanal, one of the world's largest floodplains with diverse and abundant wildlife located within the states of MS and MT. Despite the evidence of various medically important arboviruses including flaviviruses and alphaviruses in the Pantanal region, the circulation of orthobunyaviruses in the region is poorly known. The only investigation for orthobunyaviruses in the region was conducted in horses in the early 1990s [5].

Based on our previous experience exploring the prevalence of arboviruses in the Pantanal, we expected to encounter a great diversity of orthobunyaviruses there as well. Accordingly, we selected 13 Brazilian othobunyaviruses of potential medical importance for serodetection studies in a series of vertebrate specimens collected throughout the region from 2009–2011. In particular, we selected OROV, Marituba (MTBV), Guaroa (GROV), Guama (GMAV), Nepuyo (NEPUV), Murutucu (MURV), Oriboca (ORIV), Xingu (XINV), Caraparu (CARV), Catu (CATUV), Apeu (APEUV), Itaqui (ITQV) and Tucunduba (TUCV), all viruses known to cause human illness. Maguari virus (MAGV), presumably a non-medically important arbovirus that had been previously reported from Pantanal [5], was also included totaling 14 orthobunyaviruses investigated.

## Materials and methods

Equines, sheep and caimans were sampled in 17 cattle ranches visited in February and October 2009, April, September and October 2010 and January 2011 in the Nhecolândia Sub-region of Pantanal, municipality of Corumbá, MS, west-central Brazil. Equines, including horses, donkeys and mules were sampled in 15 ranches, sheep in nine and caimans in two ranches. Four equine samples collected from a ranch in Nabileque, a different sub-region of Pantanal, were also tested. Serum samples of equines, sheep and caimans, used in the present study had been previously tested for arthropod-borne viruses resulting in serological evidence of four alphaviruses and five flaviviruses [6],[7]. Considering that orthobunyaviruses are also transmitted by arthropods, a subset of sera from the same samplings was selected for the orthobunyavirus investigation.

The collections for this study were authorized by oral consent by the residents of the sampled properties after previous contact with the owners of the sampled properties. This study was approved by the Animal Ethics Committee of Fundação Oswaldo Cruz of Ministry of Health of Brazil (License CEUA-Fiocruz LW-1/12, protocol P-74/10-5) in compliance with the requirements of Brazilian Law 11794/2008, which rules on the scientific use of animals, including the principles of the Brazilian society of Science in laboratory animals. The caiman sampling was also approved by the Instituto Chico Mendes de Conservação da Biodiversidade of Ministry of Environment of Brazil (licenses ICMBio 18363-1/2009 and 18363-2/2010).

Blood samples from equines (n = 375), and from sheep (n = 232) were taken by jugular venipuncture. Information including age, health condition and travel history outside of Pantanal were recorded for each animal sampled. All the equine samples tested were seropositive for flaviviruses by blocking ELISA in a previous study indicating exposure of this subset of equines to mosquito bites [6]. Briefly, blocking ELISA evaluated the ability of the sera to block the binding of the flavivirus group-reactive monoclonal antibody 6B6C-1 to the cell lysate-derived antigen for West Nile virus (WNV) [8]. All equines and sheep were apparently healthy at time of venipuncture.

Caimans (n = 66) were captured from sites where a high concentration of these animals was observed, such as lentic systems that were formed by ephemeral rivers. Caimans were captured from boats or from the riverbanks, brought to shore and sampled by venipuncture of the internal jugular vein.

All serum samples were heat-inactivated and tested by the 90% plaque-reduction neutralization test (PRNT_90_) for their ability to neutralize plaque formation by OROV, MTBV, GROV, GMAV, NEPUV, MURV, ORIV, XINV, CARV, CATUV, APEUV, ITQV, TUCV and MAGV following standard protocols [9]. Viruses were provided by the Arbovirus Diseases Branch of the Division of Vector-Borne Diseases, Centers for Disease Control and Prevention (CDC), from its arthropod-borne virus reference collection. Low-passage preparations of the following virus strains were used in this study: OROV (TRVL9760), MTBV (BeAn15), GROV (CoH352111), GMAV (BeAn277), NEPUV (BeAn10709), MURV (BeAn974), ORIV (BeAn17), XINV (BeH388464), CARV (SPAn26550), CATUV (BeH151), APEUV (BeAn848), ITQV (BeAn12797), TUCV (BeAn278) and MAGV (BeAn7272). Mouse hyperimmune ascitic fluids (MHIAF) were used as positive control, and diluent media and serum samples of wild animals that had PRNT_90_ titers <10 for the tested viruses, as negative controls.

Briefly, in a biosafety level three facility (BSL3), serum samples were initially screened at a dilution of 1:10 and those that neutralized virus challenge by at least 90% were further tested at serial two-fold dilutions that ranged from 1:20–1:320 to determine 90% endpoint titers. Serum samples were considered seropositive in a monotypic reaction when a serum dilution of at least 1:10 reduced at least 90% of the formation of viral plaques of only one of the 14 orthobunyaviruses tested in Vero cells. Sera that presented PRNT titer ≥ 10 for more than one orthobunyavirus were considered heterotypic reactions. Serum samples with PRNT titers < 10 for all 14 orthobunyaviruses tested were considered seronegative. To save resources and considering monotypic reactions to be the most reliable with no indication of cross-reaction, for XINV that was one of the last orthobunyaviruses tested, most samples that were XINV-positive in the screening and had PRNT_90_ titers ≥10 for any other orthobunyavirus were considered heterotypic reactions and not further tested to determine XINV-endpoint titers. The same approach was used for samples that were XINV-positive in the screening and that presented PRNT_90_ titer <10 for all the other orthobunyaviruses characterizing monotypic reactions for XINV.

## Results

For 375 equines tested, 285 (76%) were heterotypic reactions with PRNT titers ≥ 10 for more than one orthobunyavirus, 69 (18.4%) showed monotypic reactions for MAGV, 19 (5.1%) were seronegative, and two (0.5%) showed monotypic reactions for XINV (Table 1). Seventeen (4.5%) equines presented heterotypic reactions for three or more orthobunyaviruses (Table 2).

**Table 1:** Monotypic reactions by PRNT_90_ for orthobunyaviruses in caimans, equines and sheep of Pantanal, Brazil.

	Free-ranging caimans (n = 66)			Equines (n = 375)			Sheep (n = 232)		
	Titer ≥ 10 (%)	Antibody titers	Number of Ranches	Titer ≥ 10 (%)	Antibody titers	Number of Ranches	Titer ≥ 10 (%)	Antibody titers	Number of Ranches
OROV	0 (0.0)		-	0 (0.0)		-	1 (0.4)	10	1 of 9
MTBV	0 (0.0)		-	0 (0.0)		-	0 (0.0)		-
GROV	0 (0.0)		-	0 (0.0)		-	1 (0.4)	20	1 of 9
GMAV	0 (0.0)		-	0 (0.0)		-	0 (0.0)		-
NEPUV	2 (3.0)	10	1 of 2	0 (0.0)		-	0 (0.0)		-
MURV	0 (0.0)		-	0 (0.0)		-	1 (0.4)	40	1 of 9
ORIV	0 (0.0)		-	0 (0.0)		-	1 (0.4)	10	1 of 9
XINV	0 (0.0)		-	2 (0.5)	≥ 10	2 of 15	14 (6)	10	7 of 9
CARV	0 (0.0)		-	0 (0.0)		-	0 (0.0)		-
CATUV	0 (0.0)		-	0 (0.0)		-	0 (0.0)		-
APEUV	0 (0.0)		-	0 (0.0)		-	3 (1.3)	10 to 20	3 of 9
ITQV	0 (0.0)		-	0 (0.0)		-	0 (0.0)		-
TUCV	0 (0.0)		-	0 (0.0)		-	0 (0.0)		-
MAGV	0 (0.0)		-	69 (18.4)	10 to 160	14 of 15	1 (0.4)	10	1 of 9

OROV: Oropouche virus, MTBV: Marituba virus, GROV: Guaroa virus, GMAV: Guama virus, NEPUV: Nepuyo virus, MURV: Murutucu virus, ORIV: Oriboca virus, XINV: Xingu virus, CARV: Caraparu virus, CATUV: Catu virus, APEUV: Apeu virus, ITQV: Itaqui virus, TUCV: Tucunduba virus, MAGV: Maguari virus.

**Table 2:** Heterotypic reactions by PRNT_90_ for more than two orthobunyaviruses in equines and sheep of Pantanal, Brazil.

ID	Species	Ranch	OROV	MAGV	XINV	MARV	MURV	APEUV	ITQV	ORIV	TUCV	GROV	GMAV	CARV	NEPUV	CATUV
384	equine	PL	<10	20	≥10	<10	<10	10	<10	<10	<10	<10	<10	<10	<10	<10
317	equine	PH	<10	160	40	<10	<10	<10	<10	<10	10	<10	<10	<10	<10	<10
578	equine	PR	<10	80	≥10	<10	<10	<10	<10	<10	10	<10	<10	<10	<10	<10
603	equine	PR	<10	20	≥10	<10	<10	10	<10	<10	<10	<10	<10	<10	<10	<10
967	equine	PC	<10	80	≥10	<10	<10	<10	<10	<10	<10	<10	<10	<10	10	<10
998	equine	PC	<10	80	≥10	<10	<10	10	<10	<10	<10	<10	<10	<10	<10	<10
845	equine	PG	<10	≥320	≥10	<10	<10	10	<10	<10	<10	<10	<10	<10	<10	<10
866	equine	PG	<10	40	≥10	<10	<10	20	<10	<10	<10	<10	<10	<10	<10	<10
868	equine	PG	<10	80	≥10	<10	10	<10	<10	<10	<10	<10	<10	<10	<10	<10
701	equine	PJ	<10	160	≥10	<10	<10	<10	<10	<10	<10	<10	<10	<10	10	<10
704	equine	PJ	<10	≥320	≥10	<10	<10	<10	<10	<10	10	<10	<10	<10	<10	<10
716	equine	PJ	<10	80	≥10	<10	<10	<10	<10	<10	10	<10	<10	<10	<10	<10
729	equine	PJ	<10	80	≥10	<10	<10	<10	<10	<10	<10	<10	<10	<10	10	<10
761	equine	PJ	<10	160	≥10	<10	<10	<10	<10	<10	20	<10	<10	<10	<10	<10
811	equine	PJ	<10	80	≥10	<10	<10	20	<10	<10	<10	<10	<10	<10	<10	<10
812	equine	PJ	<10	160	≥10	<10	<10	<10	<10	<10	10	<10	<10	<10	<10	<10
361	equine	PM	<10	80	40	<10	<10	20	<10	<10	<10	<10	<10	<10	<10	<10
23	sheep	PL	<10	160	20	<10	<10	20	<10	<10	<10	<10	<10	<10	<10	<10
38	sheep	PL	<10	≥320	≥80	<10	<10	<10	<10	<10	20	<10	<10	<10	<10	<10
158	sheep	PC	<10	≥320	≥80	<10	<10	<10	<10	<10	20	<10	<10	<10	<10	<10
166	sheep	PC	<10	≥320	≥80	<10	<10	<10	<10	<10	10	<10	<10	<10	<10	<10
103	sheep	PI	<10	80	20	<10	<10	<10	<10	<10	<10	<10	40	<10	10	<10
104	sheep	PI	<10	40	40	<10	<10	<10	<10	<10	10	<10	<10	<10	<10	<10
109	sheep	PI	<10	≥320	≥80	<10	<10	<10	<10	<10	40	<10	<10	<10	<10	<10
135	sheep	PJ	<10	≥320	≥80	<10	<10	<10	<10	<10	10	<10	<10	<10	<10	<10
1	sheep	PM	<10	≥320	≥80	<10	<10	<10	<10	<10	10	<10	<10	<10	<10	<10
6	sheep	PM	<10	≥320	≥80	<10	<10	<10	<10	<10	40	<10	<10	<10	<10	<10
7	sheep	PM	<10	160	40	<10	<10	10	<10	<10	<10	<10	<10	<10	<10	<10
17	sheep	PM	<10	80	≥80	<10	<10	10	<10	<10	<10	<10	<10	<10	<10	<10

For 232 sheep tested, 115 (49.6%) were seronegative, 95 (41%) were heterotypic reactions, 14 (6%) showed monotypic reactions for XINV, three (1.3%) for APEUV, one (0.4%) for GROV, one (0.4%) for MAGV, one (0.4%) for MURV, one (0.4%) for ORIV and one (0.4%) for OROV (Table 1). Twelve (5.2%) sheep presented heterotypic reactions for three or more orthobunyaviruses (Table 2).

For 66 free-ranging caiman samples, 63 (95%) were seronegative, two (3%) showed monotypic reactions for NEPUV and one (1.5%) was heterotypic reaction (Table 1).

## Discussion

The Pantanal, which presents vast wetland habitat in a subtropical climate, present a set of factors that supports the introduction, maintenance, and evolution of arthropod-borne viruses. The region has abundant biodiversity and is the most important waterbird breeding area in South America [10]. The Nhecolândia Sub-region of the Pantanal is the world’s largest and most biodiverse region of subtropical lakes [11],[12]. Recent arbovirus studies conducted in the region have found serological evidence of at least nine medically important arboviruses, including WNV and Mayaro virus. Moreover, Ilheus virus and six novel viruses were recently isolated from local mosquitoes [6],[7],[13],[14],[15],[16],[17].

In the absence of direct viral detection, diagnosis of arbovirus infections is performed by indirect serological tests. However, some cross-reactivity in primary infections has been reported among certain bunyaviruses. For instance, using the complement-fixation test, ORIV cross-reacted broadly with MURV antibody [18]. In neutralization tests using NEPUV and guinea pig immune sera for different group C viruses, NEPUV reacted mainly with immune serum of MURV, but also with immune sera of MARV, CARV and ITQV [19]. Additionally, antibody responses in vertebrates sequentially infected with orthobunyaviruses are not well described [20]. There is only one report that describes the antibody responses in vertebrates experimentally inoculated with two different orthobunyaviruses [21]. Moreover, other bunyaviruses may also circulate in the region, including novel orthobunyaviruses, which could theoretically generate cross-reacting neutralizing antibodies and lead to misinterpretation. A novel orthobunyavirus closely related to CARV has been recently reported in febrile patients in Peru [22]. In August of 2010, after an epizootic of illness among sylvatic monkeys, an OROV species reassortant named Madre de Dios virus was isolated from a sick monkey collected in a forest near a small rural village in Venezuela [23]. Therefore, we used a conservative threshold for detection of neutralizing antibodies (90%) in the region’s equines, sheep and wild caimans and we considered monotypic serologic responses to be the most reliable, as these samples reacted with just one of the fourteen viruses employed in the tests, with no indication of cross-reaction. This criterion may appear conservative, but the main objective is to prevent the introduction of false positives in the data, even at cost of some false negatives [24]. In the present study, monotypic reactions were encountered for local circulation of XINV, MAGV, APEUV, ORIV, OROV, MURV, NEPUV and GROV based on the detection of neutralization antibodies in equines, sheep and free-ranging caimans (S1 Data). Except for one equine that exhibited monotypic reaction to MAGV and had travel history to Sidrolândia, outside of the Pantanal, all of the seropositive animals that showed monotypic reactions, lacked travel history indicating autochthonous transmission of these orthobunyaviruses in the region.

OROV is a member of the Simbu serogoup and it has been involved in explosive human outbreaks in northern Brazil since the 1960s. Oropouche fever is characterized by an abrupt onset and fever, headache, myalgia, arthralgia, dizziness, chills and photophobia. Some cases can be severe, including neurologic disorder characterized as meningitis mainly in immunocompromised patients [25],[26]. From 2000 to 2007, OROV was detected in febrile patients from Bolivia, Ecuador and Peru [27]. Recently, OROV was reported during a dengue outbreak in MT, the west-central region of the country [4]. OROV is thought to be maintained in nature in a sylvatic cycle in which primates, sloths and birds are the amplifying hosts and the biting midge *Culicoides paraensis* is the main vector. OROV has also been isolated from mosquitoes, including *Aedes serratus* and *Culex quinquefasciatus* [26]. In the present study, only one sheep had monotypic reaction for OROV.

APEUV, CARV, ITQV, MTBV, MURV, ORIV and NEPUV are arboviruses of the group C encountered in the Amazon Basin. The group C arboviruses are maintained in nature mainly between rodents and *Culex* spp. mosquitoes. With the exception of NEPUV, all have been isolated from human beings in the Amazon Basin [26]. In the early 1970s, neutralizing antibodies for NEPUV were detected in bats from Trinidad, and the virus has been involved in human disease in Central America [28],[29]. CARV is the most widely distributed group C virus in the Amazon Basin and it causes human disease in southeast Brazil [30]. Group C arboviruses produce a febrile syndrome with sudden onset, including high fever, headache, chills, myalgia, photophobia and retrobulbar pain [31]. From 1996 to 2001, CARV, ITQV, and MURV were isolated from numerous pools of *Culex* spp. from Peru [32]. In 2001–2002, APEUV was detected in monkeys from the Brazilian Amazon [33]. CARV and MURV were also recently detected in febrile patients from Bolivia and Peru [27]. Among the group C orthobunyaviruses tested in the present study, sheep had monotypic reactions for APEUV, MURV and ORIV, and two caimans had monotypic reactions for NEPUV. All equines had heterotypic reactions or were negative for group C orthobunyaviruses.

GROV, a member of the California encephalitis serogroup, is considered one of the most widely distributed orthobunyaviruses in the Amazon region. Several strains of GROV have been isolated from febrile patients and *Anopheles* spp. mosquitoes in Brazil, and birds are suspected to be the vertebrate amplifying hosts. GROV has been involved in sporadic cases of disease with acute onset and high fever, chills, headache, myalgia and malaise in rural areas of the Amazon region [26]. GROV was recently isolated in febrile patients from Bolivia and Peru [27],[34]. In the present study, only one sheep had monotypic reaction for GROV.

GMAV and CATUV are members of the Guama serogroup and have been isolated from human blood samples in the Amazon Basin. The ecoepidemiology of these viruses is similar to the group C arboviruses [35]. Both of them have been isolated mainly from rodents and *Culex* spp. mosquitoes [26]. In the early 1970s, neutralizing antibodies for GMAV were detected in bats from Trinidad [28]. When symptomatic, infections by GMAV and CATUV have a sudden onset of mild fever, dizziness, headache, muscle pains, arthralgia, photophobia and malaise [31],[35]. All animals tested in the present study were seronegative or had heterotypic reactions for GMAV or CATUV.

TUCV and XINV are genetically characterized as members of the Wyeomyia and Bunyamwera groups, respectively [36],[37]. TUCV has been isolated from different mosquitoes, including *Wyeomyia* sp., *Sabethes* sp. and *Trichoprosopon digitatum* and was also isolated from a patient with meningoencephalitis in Brazil [38]. The vertebrate hosts of TUCV remain unknown. No monotypic reactions for TUCV were detected in the present serological inquiry. Regarding XINV, the vectors and wild vertebrate amplifiers are unknown and the virus was only isolated from a hepatitis B case with a fatal outcome in Brazil [31]. XINV was the most prevalent orthobunyavirus in Pantanal sheep. The detection of 14 (6%) sheep with monotypic reactions for XINV in seven (78%) ranches sampled suggests widespread circulation of XINV in sheep of Pantanal (Table 1). Equines with monotypic reactions for XINV were detected in two Pantanal ranches.

MAGV (Bunyamwera serogroup) was first isolated in Brazil in the 1950s from a mixed mosquito pool containing *Aedes* spp., *Mansonia* sp. and *Psorophora ferox* and since then has never been reported to cause disease. MAGV was previously classified as a subtype of Cache Valley virus, but some strains of MAGV have been shown to differ antigenically from the prototype. MAGV is now regarded as a closely related, but distinct virus [39]. The enzootic transmission cycle of MAGV is unknown, but serological evidence has been reported in birds, sheep, water buffalo, man, cattle and mainly horses, from which MAGV has been isolated in Colombia and Guyana [39]. An arbovirus investigation conducted also in the Pantanal in the 1990s detected serological evidence for MAGV in 28% of the equines tested [5]. In the present study, one sheep and 69 (18.4%) equines from 14 (93%) ranches had monotypic reactions for MAGV. Together, these studies suggest that the circulation of MAGV in equines from Pantanal has been active for at least three decades and that MAGV is now widely distributed in the region (Table 1). Evidence reported here also suggest current or recent circulation of MAGV in the Pantanal. Among the equines with monotypic reactions for MAGV, three animals were two-year-old at the moment of the venopuncture in 2009 suggesting that MAGV circulated in equines of the region between 2007 and September 2009.

Except for MAGV, which was previously detected in horses in the 1990s [5], the detection of monotypic reactions for seven other orthobunyaviruses provides the first evidence of their circulation in the Pantanal. Considering the potential for cross-reaction complicates the interpretation of serological tests, more investigation is needed to confirm their circulation by virus isolation, to determine the public health burden and understand the ecology of transmission of these viruses in the Pantanal region, including identifying amplifier hosts and vectors.

The biased selection of equines for our study (positive by blocking ELISA for flaviviruses) may have contributed to our high prevalence for MAGV result in equines. If so, an explanation is that the vector or vertebrate hosts involved in transmitting or amplifying flaviviruses may play a similar role for MAGV.

The vertebrate amplifying hosts of these viruses in the Pantanal have not been studied and testing only equines, sheep and caimans may provide a biased view of the relative amounts of orthobunyavirus transmission because these hosts may not attract all vectors equally. In the present study, there was a significant difference in the number of orthobunyaviruses detected according to the host tested. For instance, sheep had monotypic reactions for seven orthobunyaviruses, while equines only for two. Sheep may attract more orthobunyavirus vectors than equines in the region and may be useful surrogates for detection of orthobunyavirus activity in the Pantanal. Interestingly, the same is not true for flaviviruses. A previous study reported that equines are more exposed to flaviviruses than sheep in the region [6]. A serosurvey of free-ranging rodents, as well as, non-human primates and/or local human residents would be interesting as an additional investigational tool for some of these orthobunyaviruses. In fact, a recent survey conducted among free-living non-human primates in MS outside the boundaries of the Pantanal found one animal with OROV-reactive hemagglutination-inhibiting antibodies [40].

Caimans were selected for inclusion in our study because reptiles may play a larger role in the transmission cycle of arboviruses than previously assumed [41]. However, we found that only two caimans were seropositive for NEPUV, suggesting that the participation of caimans in other orthobunyavirus transmission cycles in the Pantanal is unlikely. However, interestingly monotypic reactions for NEPUV were detected only in caimans.

The prevalence of MAGV in equines and XINV in sheep suggests that both viruses are widespread in the Pantanal. Another explanation would be cross-reaction between XINV and MAGV, which are considered indistinguishable by some classical tests [26]. Both viruses are members of the Bunyamwera serogroup, but the molecular characterization of XINV confirms them as different orthobunyaviruses [36]. In fact, the number of MAGV-seropositive equines in the present study was higher than the number of XINV-seropositive equines. Thirty animals, including equines and sheep, presented heterotypic reactions not only for both MAGV and XINV, but also for a third orthobunyavirus, including TUCV (n = 14), APEUV (n = 10), NEPUV (n = 4), GMAV (n = 1), MURV (n = 1) and ORIV (n = 1). One sheep presented neutralizing antibodies to four orthobunyaviruses (Table 2). Together these findings might indicate either cross-reactivity among the orthobunyaviruses tested or multiple exposure of Pantanal animals to various orthobunyaviruses throughout their lifetimes.

Conservative serologic criteria were used to present evidence of local circulation of orthobunyaviruses primarily in equines, but also in sheep and caimans in the Pantanal, Brazil. The detection of seropositive animals for seven medically important orthobunyaviruses are novel findings for the Pantanal. However, because detection of antibodies is indirect evidence of virus circulation and because unknown orthobunyaviruses may circulate in the region, we encourage efforts to isolate viruses to confirm the circulation of these orthobunyaviruses in the Pantanal. Despite the low titers for most of the orthobunyaviruses tested, monotypic reactions for eight orthobunyaviruses suggest the Pantanal as a region of great orthobunyavirus diversity. The present data, in conjunction with previous studies that detected local circulation of various flaviviruses and alphaviruses, confirm the Pantanal as an important natural reservoir for zoonotic arboviruses in Brazil.

## Supporting information

S1 Data PRNT_90_ titers for orthobunyaviruses in equines, sheep and free-ranging caimans from Pantanal, Brazil Legend: H—Heterotypic reaction; N—Seronegative; XINV—Xingu virus; OROV—Oropouche virus; ORIV—Oriboca virus; MURV—Murutucu virus; MAGV—Maguari virus; GROV—Guaroa virus; APEUV—Apeu virus; NEPUV—Nepuyo virus. (XLSX)
